# Emerging Digital Technologies for Monitoring and Rehabilitation Support in Chronic Dust-Induced Lung Diseases: A Scoping Review

**DOI:** 10.3390/jpm16080407

**Published:** 2026-07-29

**Authors:** Nazym Sagandykova, Madina Baurzhan, Alexandr Gulyayev, Sayagul Kairgeldina, Shyngys Sergazy, Gulnur Daniyarova, Karlygash Absattarova, Liudmila Kovalenko, Akmaral Izbassarova

**Affiliations:** 1 Department of Internal Diseases, Corporate Fund “University Medical Center”, Astana 010000, Kazakhstan; doctor.ent.alm@gmail.com; 2 Research Institute of Balneology and Medical Rehabilitation of the Ministry of Health of the Republic of Kazakhstan, Astana 010000, Kazakhstan; akin@mail.ru (A.G.); sanborovoe@mail.kz (S.K.); shynggys.sergazy@nu.edu.kz (S.S.); gulnurdaniyarkz@gmail.com (G.D.); absatarova.k@kaznmu.kz (K.A.); 3 Laboratory of Drug Discovery and Development, Nazarbayev University, Astana 010000, Kazakhstan; 4 Department of Clinical Pharmacology and Evidence-Based Medicine, JSC “Karaganda Medical University”, Karaganda 100008, Kazakhstan; 5 Department of Physical Medicine and Rehabilitation, Kazakh National Medical University Named After S.D. Asfendiyarov, Almaty 050012, Kazakhstan; aksha5@mail.ru; 6 Department of Pathophysiology, Surgut State University, Surgut 628403, Russia; kovalenko_lv@surgu.ru

**Keywords:** chronic dust-induced lung diseases, pneumoconiosis, wearable devices, digital monitoring, machine learning, rehabilitation

## Abstract

**Background/Objectives:** Chronic dust-induced lung diseases, including pneumoconiosis and asbestosis, require long-term monitoring and individualized management. Conventional rehabilitation and follow-up often depend on in-person visits and may not provide continuous assessment outside clinical settings. This scoping review mapped evidence on digital technologies used to monitor patients, assess respiratory symptoms and functional status, and support rehabilitation follow-up in chronic dust-induced lung diseases. **Methods:** The review followed the Preferred Reporting Items for Systematic Reviews and Meta-Analyses extension for Scoping Reviews (PRISMA-ScR) and Joanna Briggs Institute methodology. PubMed, Scopus, and Web of Science were searched from inception to 29 June 2026 without language restrictions. Five eligible publications were included. **Results:** The evidence was grouped into three categories: wearable monitoring of activity and respiratory symptoms, analysis of data from a digital rehabilitation platform, and electronic medical record (EMR) analysis. Wearable sensors showed high performance in recognizing basic activity states and cough events under controlled conditions. Platform- and EMR-based approaches showed potential for using clinical and functional data to support patient stratification and monitoring. However, most studies were limited to early technical or algorithmic validation and did not assess long-term home use, patient adherence, integration into clinical workflows, or clinical and rehabilitation outcomes. **Conclusions:** Digital technologies may support objective monitoring and rehabilitation follow-up in chronic dust-induced lung diseases, but the evidence base remains small and clinically immature. Prospective studies in real-world settings should evaluate usability, external validity, workflow integration, and clinically meaningful outcomes.

## 1. Introduction

Occupational lung diseases caused by industrial dust exposure remain a substantial and largely preventable global health problem. Occupational interstitial lung diseases, including silicosis, asbestosis, and other pneumoconioses, continue to occur in mining, construction, metallurgy, and other dust-generating industries despite advances in preventive measures [1,2]. This problem is of particular importance for countries with large extractive and industrial sectors, as chronic dust-related lung diseases lead to long-term disability, the need for long-term clinical monitoring, and an increased social burden. This problem is further emphasized by the fact that silicosis and other forms of pneumoconiosis remain a serious occupational hazard in countries with expanding industrial sectors and require systematic programs for prevention, early detection, and long-term patient management [3].

The clinical significance of chronic dust-induced lung diseases extends beyond diagnosis. These diseases are often progressive and are characterized by chronic pulmonary inflammation and irreversible fibrotic changes. Because effective disease-modifying treatment remains limited, long-term management primarily focuses on preventing further exposure, controlling symptoms, monitoring functional status, preventing complications, and providing rehabilitation support [4,5,6]. In this context, pulmonary rehabilitation and patient education become important components of pneumoconiosis management, as they can contribute to improving functional status, exercise tolerance, self-management, and quality of life [7,8].

However, traditional models of follow-up and rehabilitation often depend on regular visits to specialized occupational medicine or pulmonology centers. For patients living in industrial and remote regions, access to specialized follow-up and rehabilitation services may be limited by geographical, logistical, and organizational barriers [9,10]. In the broader field of chronic disease, digital health interventions, telemedicine approaches, and remote rehabilitation formats are increasingly being explored as ways to improve access, personalization, and continuity of care [11,12,13,14,15]. These findings provide a rationale for evaluating similar approaches in chronic dust-induced lung diseases; however, disease-specific evidence has not yet been systematically mapped.

The purpose of this scoping review is to examine the available evidence on digital technologies used to monitor patients with chronic dust-induced lung diseases, assess respiratory symptoms and functional indicators, and support rehabilitation follow- up. The review aims to map the types of technologies used, the patient groups studied, the clinical, functional, and technical outcomes presented, and determine the extent to which these technologies have progressed beyond technical or algorithmic validation toward clinical implementation.

## 2. Materials and Methods

This scoping review was conducted in accordance with the PRISMA-ScR (Preferred Reporting Items for Systematic Reviews and Meta-Analyses extension for Scoping Reviews) checklist [16] and the Joanna Briggs Institute (JBI) methodology for scoping reviews [17]. The study protocol was registered on the Open Science Framework (OSF; https://doi.org/10.17605/OSF.IO/HXYNG).

The review addressed the following question: what digital technologies are used for monitoring patients with chronic dust-induced lung diseases, assessing respiratory symptoms and functional parameters, and supporting rehabilitation follow-up.

Research question: What digital technologies have been used or described for monitoring and rehabilitation follow-up of patients with chronic dust-induced lung diseases, and what clinical, functional, or technical results of their use have been presented in the scientific literature?

The literature search was conducted in PubMed, Scopus, and Web of Science from database inception to 29 June 2026, without restrictions on language or year of publication. The search was performed by N.S. and G.D., and the search strategies and retrieved records were verified by A.G. and M.B. Two reviewers (S.K. and Sh.S.) independently screened titles and abstracts and subsequently assessed potentially eligible full texts. Disagreements were resolved through discussion or consultation with a third reviewer (A.I.).

The search was organized into two main streams: digital monitoring and digital rehabilitation. This approach reflected differences in terminology between digital monitoring technologies and rehabilitation-related technologies and was intended to maximize search sensitivity. The full search strategies, including search terms, database-specific syntax, and the number of records retrieved from each database, are provided in Supplementary Table S2.

The review included original studies involving patients with confirmed chronic non-malignant dust-induced lung diseases, including silicosis, asbestosis, coal workers’ pneumoconiosis, mixed-dust pneumoconiosis, and other non-malignant pneumoconioses. Studies were eligible if they described digital technologies used for patient monitoring, assessment of respiratory symptoms or functional parameters, rehabilitation-related data collection, rehabilitation support, or analysis of patient-level digital data relevant to monitoring or rehabilitation follow-up in this patient population. Eligible study types included clinical and observational studies, pilot and technical-validation studies, retrospective model-development studies, qualitative and mixed-methods studies, and full-length conference papers with sufficient methodological and results information.

Studies were excluded if they involved only healthy dust-exposed workers without an established dust-induced lung disease, focused solely on environmental dust exposure monitoring, addressed only initial diagnosis or occupational risk prediction without patient-level monitoring or rehabilitation relevance, or evaluated non-digital interventions. Reviews, editorials, letters, research protocols, and conference abstracts without available full-text descriptions of methods and results were also excluded. Studies with mixed populations were included only when data on patients with dust-induced lung diseases could be extracted separately.

Records without abstracts were not automatically excluded. If eligibility could not be determined from the title alone, the full text was sought and assessed.

All records retrieved from PubMed, Scopus, and Web of Science were exported and imported into Rayyan (Rayyan Systems Inc., Cambridge, MA, USA) [18] for duplicate detection, title and abstract screening, and full-text eligibility assessment. After removal of duplicates, records were screened against the predefined eligibility criteria. Records with titles or abstracts in non-English languages were not excluded on the basis of language; available English metadata was used when present, and machine translation was used when needed to assess eligibility. Full texts of potentially relevant articles were then reviewed in detail, and reasons for exclusion at the full-text stage were documented.

Data from the included studies were charted using a structured form in Rayyan. We collected information on publication details, study population, disease type, digital technology, study design, analytical method, main outcomes, limitations, and relevance to monitoring or rehabilitation follow-up. We also noted whether studies used independent datasets or were related to other included publications.

Because the included studies were few and heterogeneous, meta-analysis was not appropriate. The findings were summarized narratively and grouped according to the main function of the digital technology: wearable activity monitoring, wearable symptom monitoring, rehabilitation-platform data analysis, and EMR-based data analysis.

A formal risk-of-bias assessment was not performed, as the purpose of this scoping review was to map the available evidence rather than estimate intervention effectiveness. However, key methodological limitations of each study were extracted and considered during the synthesis.

## 3. Results

**Figure 1:**
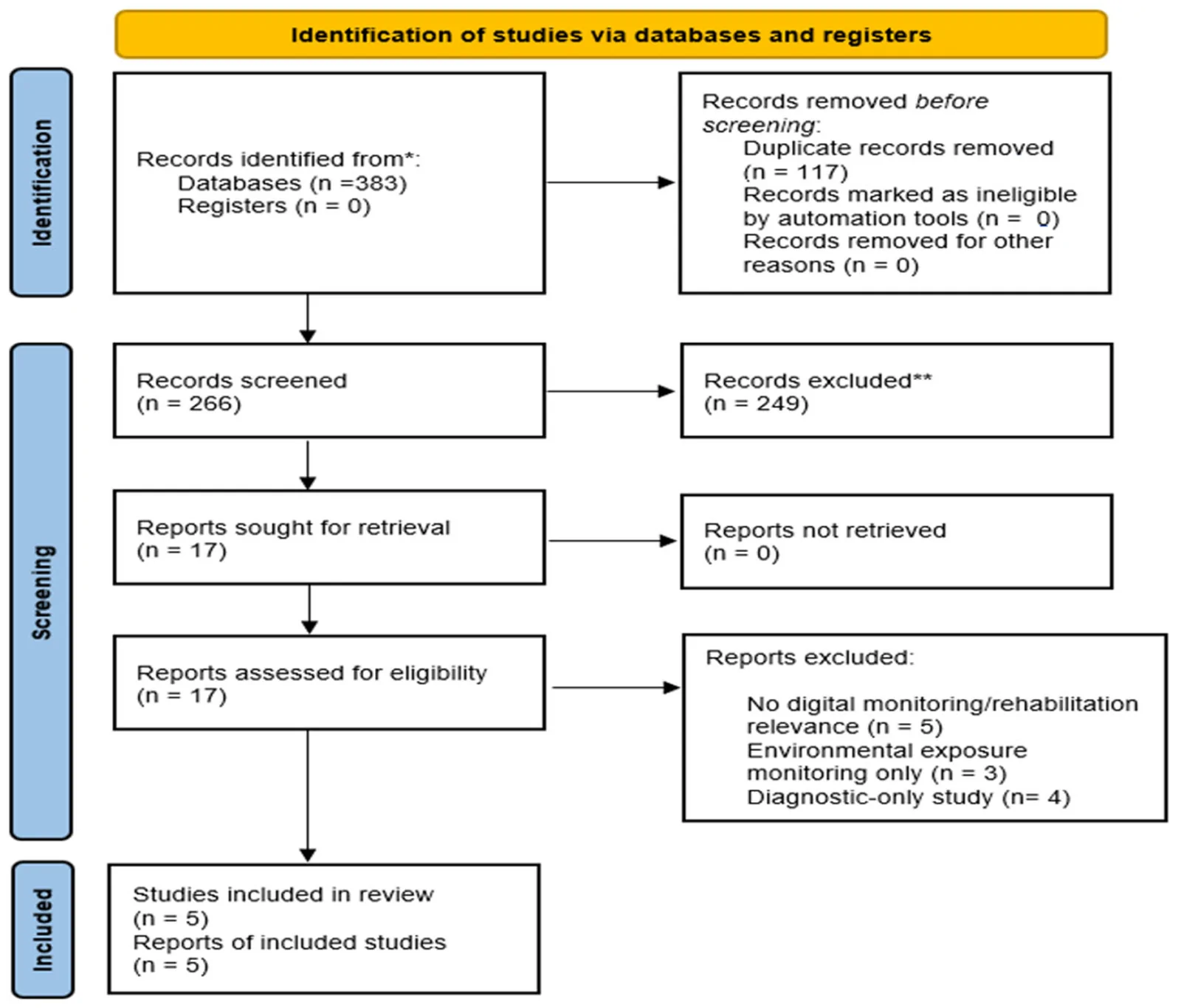
PRISMA-ScR diagram of the study-selection process. * The records were identified through searches of PubMed, Scopus, and Web of Science. ** Records were excluded after title and abstract screening based on the predefined inclusion and exclusion criteria.

The database search identified 383 records. After removal of 117 duplicate records, 266 records were screened by title and abstract. Of these, 249 records were excluded as not meeting the eligibility criteria. The full texts of 17 articles were assessed for eligibility, and 12 articles were excluded with reasons. Finally, five publications met the inclusion criteria and were included in the scoping review. The study selection process is shown in Figure 1.

The included publications were grouped into three thematic categories based on the primary function of the digital technology: wearable activity and respiratory symptom monitoring, digital rehabilitation platform data analysis, and electronic medical record analysis. This structure was used to summarize the data by technology function rather than by publication type, as the included studies varied in design, data sources, and level of clinical maturity. The main characteristics of the included studies are presented in Table 1.

### 3.1. Wearable-Based Monitoring of Activity and Respiratory Symptoms

Three publications focused on the use of wearable sensor technologies for monitoring physical activity and respiratory symptoms in patients with pneumoconiosis. Ren et al. [23] evaluated a flexible chest patch combining a triaxial accelerometer and single-channel electrocardiography (ECG) for recognizing basic motor states, including sitting, standing, lying down, and walking. The convolutional neural network–long short-term memory (CNN–LSTM) model achieved the highest performance. Using combined accelerometry and ECG data, it classified basic activity states in patients with pneumoconiosis with an accuracy of 94.25%. The findings demonstrate the technical feasibility of wearable physical activity monitoring; however, they reflect the algorithm’s performance under controlled conditions rather than confirming the system’s clinical effectiveness in real-world rehabilitation practice.

Mekruksavanich et al. [22] reanalyzed the same Clinical Human Activity Recognition (Clinical HAR) dataset using deep learning models, including CNN, LSTM, bidirectional long short-term memory (BiLSTM), gated recurrent unit (GRU), and bidirectional gated recurrent unit (BiGRU). Based on five-fold cross-validation, the BiGRU model demonstrated the best performance in the pneumoconiosis patient group: activity recognition accuracy was 97.14 ± 0.20%, F1-score was 97.20 ± 0.20%, and loss function value was 0.10 ± 0.01. In comparison, the accuracies of the GRU, CNN, and LSTM models were 96.64%, 93.20%, and 80.47%, respectively. Within this dataset, the BiGRU model yielded the highest reported performance; however, this work should be considered a secondary analysis of the previously published dataset by Ren et al., rather than an independent clinical validation.

In a separate study, Wang et al. [21] developed an automatic cough recognition system based on a flexible chest patch with a triaxial accelerometer. The study included 25 patients with pneumoconiosis and 25 healthy volunteers; accelerometry signals were analyzed using several machine learning algorithms. The Random Forest model demonstrated the best results in the patient group: cough recognition accuracy was 96.1%, precision was 95.0%, recall was 97.4%, F1-score was 96.2%, and area under the curve (AUC) = 98.7%. These results demonstrate high algorithmic accuracy of cough recognition under controlled testing conditions.

Overall, wearable sensor technologies have demonstrated the potential for objectively recognizing physical activity and cough in patients with pneumoconiosis. However, all three publications represented early technical or algorithmic validation. The main limitations included small sample sizes, standardized data-collection conditions, absence of external validation and long-term home monitoring, and no assessment of adherence, clinical deterioration, hospitalization, or rehabilitation outcomes. Therefore, the available data support the technical feasibility of wearable monitoring but do not yet demonstrate its clinical effectiveness.

### 3.2. Platform-Based Rehabilitation Data Analysis

One publication analyzed data from a digital rehabilitation platform for patients with pneumoconiosis [20]. Zhang et al. developed an eXtreme gradient boosting (XGBoost) model based on data from the ‘’Chongqing 5G Pneumoconiosis Rehabilitation Management Information Platform’’. The retrospective study included 4220 patients with occupational pneumoconiosis undergoing rehabilitation; of these, 1872 patients had complications and 2348 were uncomplicated.

The study’s methodological basis was to compare the complication status of pneumoconiosis patients with clinical and functional indicators collected in the digital rehabilitation platform. After feature selection, the model included age, type and stage of pneumoconiosis, activities of daily living (ADL), muscle strength, dyspnea severity according to mMRC (modified medical research council), cough, sputum viscosity, New York Heart Association (NYHA) functional class, 6 min walk test, and the Borg scale. The model demonstrated moderate discriminatory ability: AUC was 0.718, accuracy was 0.666, sensitivity was 0.547, specificity was 0.763, F1-score was 0.622, and Brier score was 0.211. SHapley Additive exPlanations (SHAP) analysis revealed that the most influential features were the Borg scale score, age, 6 min walk test distance, muscle strength, disease stage, and cough.

From a digital rehabilitation perspective, this study demonstrates that data routinely collected within a rehabilitation platform can be used not only for documenting patient status but also for risk-based monitoring. Such a model could potentially help identify patients who require more frequent monitoring of symptoms, functional status, and tolerance of rehabilitation interventions. However, the results should be interpreted with caution: the study was retrospective, the model was only internally validated, and its impact on real clinical decisions, rehabilitation dynamics, complication rates, or hospitalizations has not yet been assessed.

### 3.3. Electronic Medical Record-Based Patient Data Analysis

One publication focused on the analysis of electronic medical records of patients with asbestosis [19]. Song et al. proposed the Un-Apriori algorithm for extracting association rules from unstructured electronic medical records. The authors used inpatient EMR data from patients with pulmonary asbestosis over a 4-year period and developed a preprocessing procedure to transform complaints, symptoms, complications, vital signs, and hospitalization intervals into categorical features for Apriori analysis.

The authors performed patient-specific and population-level analyses. The identified rules linked symptoms, comorbidities, readmission intervals, and signs of potential deterioration. The authors viewed these data as a basis for supporting personalized management and daily monitoring of patients with asbestosis.

However, the study represented preliminary algorithmic development. The sample size was poorly described, much of the data preprocessing was manual. Neither external nor clinical validation was performed, and the system was not prospectively implemented for real-world patient monitoring. Therefore, this work should be viewed as an early example of EMR data analysis potentially useful for supporting patient monitoring, but not as proof of the effectiveness of digital monitoring or rehabilitation.

## 4. Discussion

### 4.1. Principal Findings and Clinical Contribution

The main finding of this scoping review is that digital technologies for monitoring and rehabilitative care for patients with chronic dust-induced lung diseases are still in their early stages of development: the final synthesis included only five publications, primarily focused on technical or algorithmic validation. The included studies demonstrate that wearable sensors can detect physical activity and cough in patients with pneumoconiosis, and data from digital rehabilitation platforms and electronic medical records were used to characterize functional status, symptoms, and recorded complication status. To our knowledge, this is the first review to map patient-level digital technologies for monitoring and rehabilitative follow- up for chronic dust-induced lung diseases, rather than for chronic respiratory diseases in general. However, the review highlights a key gap between technical feasibility and proven clinical effectiveness: existing studies demonstrate the potential of digital measurements and algorithms, but do not yet show that these technologies improve rehabilitation outcomes, clinical decision- making, patient adherence, or early detection of deterioration in real-world practice.

### 4.2. Methodological Modalities and Interpretability

Methodologically, the included publications varied significantly in the type of digital data and analytical approaches. Studies on wearable monitoring used accelerometry signals, alone or in combination with ECG, which were analyzed using machine and deep learning algorithms, including Random Forest, CNN-LSTM, GRU, and BiGRU. These models demonstrated high accuracy in recognizing physical activity and cough, but their interpretability was limited, particularly in the deep learning studies, which focused on algorithmic performance rather than explaining clinically relevant features. In contrast, studies based on a digital rehabilitation platform and electronic medical records were closer to clinically interpretable analysis: the XGBoost model used functional and symptomatic measures such as mMRC, 6 min walk test, Borg scale, muscle strength, and cough, while SHAP analysis allowed for the assessment of the contribution of individual features. An EMR-based approach using association rules also demonstrated interpretability, identifying links between symptoms, comorbidities, and hospital readmissions. Thus, existing studies demonstrate a wide range of methodological modalities—from sensory signals to platform and EMR data—but an important methodological gap remains: future digital systems for monitoring and rehabilitation support must combine high algorithmic accuracy with clinical explainability, external validation, and testing of applicability in real-world monitoring settings.

### 4.3. Resource Distribution, Data Heterogeneity, and Feasibility

The implementation of digital monitoring and rehabilitation support for chronic dust-induced lung diseases should be considered not only as a technical but also as an organizational challenge. On the one hand, such technologies are potentially particularly important for this group of patients, since access to specialized screening, occupational health care, and pulmonary rehabilitation is often limited by territorial, personnel, and infrastructural barriers. This is confirmed by studies on mobile clinics and telemedicine screening in miners [24] and studies of barriers to pulmonary rehabilitation in chronic respiratory diseases [9,10,25]. On the other hand, the translation of digital solutions into real-world practice is complicated by data heterogeneity: wearable sensors, rehabilitation platforms, and electronic health records generate different types of data that vary in format, collection frequency, completeness, quality, and clinical interpretability. In this regard, issues of EHR semantic interoperability, standardization of digital outcomes, and integration of wearable-derived data with clinical data become critical for future research [26,27]. Furthermore, evidence from the broader field of chronic respiratory diseases suggests that telerehabilitation and remote respiratory assessment can be feasible and potentially useful, but their effectiveness depends on patient adherence, digital literacy, stability of data transmission, acceptance of the technology by healthcare professionals, and the availability of technical support [11,28,29,30,31]. Therefore, for patients with pneumoconiosis, silicosis, and asbestosis, future digital solutions should be evaluated not only for the accuracy of algorithms but also for their practical applicability in settings with uneven access to care, heterogeneous data sources, and limited healthcare resources.

### 4.4. Comparison with Extant Literature

The findings of this review are consistent with broader literature showing increasing interest in digital health, telerehabilitation, and wearable monitoring in chronic respiratory diseases. Recent systematic reviews have suggested that digital interventions, telerehabilitation, and wearable devices may support symptom monitoring, physical activity assessment, pulmonary rehabilitation, and continuity of care in COPD and other chronic respiratory disease populations [11,12,13]. Qualitative evidence also indicates that the usefulness of activity monitors depends not only on measurement accuracy, but also on usability, feedback, motivation, and integration into patients’ daily self-management [32]. However, our review shows that evidence specific to chronic dust-induced lung diseases remains substantially more limited. In contrast to the broader respiratory literature, the included studies in pneumoconiosis and asbestosis mainly demonstrate technical or algorithmic feasibility, such as recognition of activity and cough or analysis of platform and EMR data, rather than validated clinical effectiveness. Thus, the present review refines existing evidence by identifying a disease-specific gap: digital tools are conceptually relevant for monitoring and rehabilitation follow-up in dust-induced lung diseases, but their real-world clinical value has not yet been established.

### 4.5. Limitations

This scoping review has several limitations. First, despite a broad search across three databases without restrictions on publication year or language, only a small number of eligible publications were identified, reflecting the limited evidence base in this field. Second, the included studies were heterogeneous in design, population, technology type, data source, analytical approach, and reported outcomes, which precluded quantitative synthesis. Third, part of the evidence came from conference proceedings or secondary algorithmic analyses, and one wearable activity-recognition publication used a dataset that overlapped with a previously published study. Fourth, most included studies were early-stage technical or retrospective model-development studies conducted in controlled or non-prospective settings; therefore, their findings cannot be interpreted as evidence of clinical effectiveness. Finally, as this was a scoping review aimed at mapping available evidence, formal risk-of-bias assessment was not performed. However, key methodological limitations of individual studies were extracted and taken into account during the descriptive synthesis and interpretation of the results.

## 5. Conclusions

This scoping review shows that digital technologies for monitoring and rehabilitation follow-up in chronic dust-induced lung diseases remain at an early stage of development. The available evidence is limited to a small number of heterogeneous publications, mainly focused on technical or algorithmic feasibility rather than clinical effectiveness. Wearable sensors, rehabilitation-platform data, and EMR-based analysis may support objective assessment of physical activity, respiratory symptoms, functional status, and risk profiles in patients with pneumoconiosis and asbestosis. However, current studies do not establish that these technologies improve rehabilitation outcomes, support clinical decision-making, sustain patient adherence, or enable earlier detection of deterioration during long-term real-world monitoring. Future research should therefore prioritize prospective, disease-specific studies that evaluate not only algorithm performance, but also usability, integration into clinical workflows, and clinically meaningful outcomes.

## Figures and Tables

**Table 1:** Characteristics, principal findings, and clinical maturity of the included publications.

Study	Publication Type and Purpose of the Study	Population and Disease	Digital Technology/Data Source	Study Design	Analytical Method	Main Findings	Main Limitations	Clinical Maturity and Relationship
B. Song, Y. Feng, X. Li, Z. Sun and Y. Yang, “Un-apriori: A novel association rule mining algorithm for unstructured EMRs,” 2017 IEEE 19th International Conference on e-Health Networking, Applications and Services (Healthcom), Dalian, China, 2017, pp. 1–6, https://doi.org/10.1109/HealthCom.2017.8210792 [19]	Conference proceeding To identify symptom patterns for decision support and daily disease management	Patients with pulmonary asbestosis from inpatient EMRs over 4 years; exact sample size not clearly reported	Unstructured electronic medical records; Un-Apriori association rule mining algorithm	EMR-based algorithm development	Un-Apriori based on Apriori after EMR preprocessing	Association rules between symptoms, complications, vital signs and hospitalization intervals	Preliminary study; manual preprocessing; no external/prospective validation; no real-time monitoring; no clinical outcome assessment	EMR-based decision support—preliminary algorithm development Independent study; not a rehabilitation intervention
ZHANG Li, PENG Peng, WANG Yun, LUO Dong. Identification of risk factors for pneumoconiosis-related complications and development and application of an XGBoost-based early prediction model. Journal of Environmental and Occupational Medicine, 2026, 43(3): 302–310. https://doi.org/10.11836/JEOM25348 [20]	Journal article To stratify risk of pneumoconiosis-related complications	4220 patients with occupational pneumoconiosis; 1872 with complications, 2348 without	Chongqing 5G Pneumoconiosis Rehabilitation Management Information Platform	Retrospective model development	LASSO/logistic regression, XGBoost, SHAP	AUC 0.718; accuracy 0.666; sensitivity 0.547; specificity 0.763; key predictors included Borg score, age, 6MWT, muscle strength, stage and cough	Retrospective; internal validation only; no prospective testing; classifies existing complications rather than true future worsening	Digital risk stratification— retrospective model development Independent study; platform-based clinical data
Wang, J., Min, C., Yu, F. et al. Cough recognition in pneumoconiosis patients based on a flexible patch with an embedded ACC sensor for remote monitoring. BMC Med Inform Decis Mak 25, 41 (2025). https://doi.org/10.1186/s12911-025-02879-y [21]	Journal article To detect cough episodes for potential remote symptom monitoring	25 patients with pneumoconiosis and 25 healthy volunteers	Flexible chest patch with tri-axial accelerometer	Controlled technical validation	Feature extraction; Random Forest and other machine-learning classifiers	Accuracy 96.1%; precision 95.0%; recall 97.4%; F1 96.2%; AUC 98.7% in patients	Small sample; controlled setting; mostly prompted cough; no home monitoring; no clinical worsening outcomes; no external validation	Wearable symptom monitoring—proof-of-concept/early technical validation Possible population overlap with Ren et al.; different task and dataset
S. Mekruksavanich, P. Jantawong and A. Jitpattanakul, “Enhancing Clinical Activity Recognition with Bidirectional RNNs and Accelerometer-ECG Fusion,” 2024 21st International Conference on Electrical Engineering/Electronics, Computer, Telecommunications and Information Technology (ECTI-CON), Khon Kaen, Thailand, 2024, pp. 1–4, https://doi.org/10.1109/ECTI-CON60892.2024.10594977 [22]	Conference proceeding To improve activity recognition using deep learning	Secondary analysis of Clinical HAR dataset: 25 pneumoconiosis patients and 20 healthy adults	Public Clinical HAR dataset with wearable ACC–ECG data	Secondary algorithmic analysis	CNN, LSTM, BiLSTM, GRU, BiGRU	BiGRU achieved highest activity-recognition performance; patient accuracy about 97.14%, F1 about 97.20%	No new clinical data; same dataset as Ren et al.; controlled activities; no home monitoring; no clinical outcomes	Wearable activity monitoring—secondary algorithmic validation Same dataset as Ren et al.; should not be counted as independent clinical population
Ren Y, Liu M, Yang Y, Mao L, Chen K. Clinical human activity recognition based on a wearable patch of combined tri-axial ACC and ECG sensors. Digit Health. 2024 Jan 4;10:20552076231223804. https://doi.org/10.1177/20552076231223804. PMID: 38188858; PMCID: PMC10768627 [23]	Journal article To recognize physical activity for potential functional monitoring and rehabilitation assessment	25 patients with pneumoconiosis and 20 healthy controls	Wearable chest patch with tri-axial accelerometer and single-lead ECG	Controlled technical validation	CNN, LSTM, CNN–LSTM, GRU	CNN–LSTM with ACC+ECG achieved accuracy 94.25% in patients	Small sample; controlled setting; short scripted activities; no longitudinal home monitoring; no rehab outcomes; no external validation	Wearable activity monitoring—early technical validation Original Clinical HAR dataset; related to Mekruksavanich secondary analysis

## Data Availability

No new data were created or analyzed in this study. All data supporting the findings of this review are contained within the article and its Supplementary Materials.

## Supplementary Materials

The following supporting information can be downloaded at: https://www.mdpi.com/article/10.3390/jpm16080407/s1, Table S1: Full search strategies and number of records retrieved from each database; Table S2: PRISMA Checklist.

## Abbreviations

The following abbreviations are used in this manuscript:

ADL	activities of daily living
AUC	area under the receiver operating characteristic curve
BiGRU	bidirectional gated recurrent unit
BiLSTM	bidirectional long short-term memory
CNN	convolutional neural network
ECG	electrocardiography
EMR	electronic medical record
GRU	gated recurrent unit
HAR	human activity recognition
JBI	Joanna Briggs Institute
