# Supplementary material for: Emerging Digital Technologies for Monitoring and Rehabilitation Support in Chronic Dust-Induced Lung Diseases: A Scoping Review
Source: J Pers Med. 2026 Jul 29;16(8):407. doi: 10.3390/jpm16080407 (PMC13514195; doi:10.3390/jpm16080407)
Supplement: Supplementary file 1 [file jpm-16-00407-s001.zip › Table S1.pdf]

Supplementary Table S1. Full search strategies and number of records retrieved from each database

Database	Search Query (Keywords)	Filters Applied	Results
PubMed	("Pneumoconiosis"[Mesh] OR pneumoconiosis[tiab] OR silicosis[tiab] OR asbestosis[tiab] OR "coal workers' pneumoconiosis"[tiab] OR "coal worker pneumoconiosis"[tiab] OR "mixed dust pneumoconiosis"[tiab] OR "dust-induced lung disease*"[tiab] OR "dust-related lung disease*"[tiab]) AND (telemonitor*[tiab] OR "remote monitor*"[tiab] OR "continuous monitor*"[tiab] OR wearable*[tiab] OR sensor*[tiab] OR telemedicine[tiab] OR telehealth[tiab] OR "digital health"[tiab] OR "home spirometr*"[tiab] OR oximetr*[tiab] OR "mobile app"[tiab] OR "mobile apps"[tiab] OR "mobile application*"[tiab] OR IoT[tiab])	No filters; search date: 29 June 2026	137
	("Pneumoconiosis"[Mesh] OR pneumoconiosis[tiab] OR silicosis[tiab] OR asbestosis[tiab] OR "dust induced lung disease*"[tiab] OR "dust related lung disease*"[tiab]) AND (rehabilitat*[tiab] OR telerehabilitat*[tiab] OR "pulmonary rehabilitation"[tiab] OR "home based rehabilitation"[tiab] OR "exercise training"[tiab] OR "physical training"[tiab] OR "respiratory training"[tiab])	No filters; search date: 29 June 2026	13

Database	Search Query (Keywords)	Filters Applied	Results
	AND<br>(digital[tiab] OR remote[tiab] OR telehealth[tiab] OR telemedicine[tiab] OR wearable*[tiab] OR mobile[tiab] OR sensor*[tiab] OR application*[tiab] OR smartphone*[tiab] OR "web based"[tiab] OR "technology assisted"[tiab])		
Web of Science	Topic: pneumoconiosis OR silicosis OR asbestosis OR "coal workers pneumoconiosis" OR "coal worker pneumoconiosis" OR "mixed dust pneumoconiosis" OR "dust induced lung disease*" OR "dust related lung disease*"<br>AND<br>Topic: telemonitoring OR "remote monitoring" OR wearable OR sensor* OR telemedicine OR telehealth OR "digital health" OR "home spirometry" OR oximetry OR "mobile app" OR "mobile apps" OR "mobile application*" OR IoT	No filters; search date: 29 June 2026	57
	TOPIC: pneumoconiosis OR silicosis OR asbestosis OR "coal workers pneumoconiosis" OR "coal worker pneumoconiosis" OR "mixed dust pneumoconiosis" OR "dust induced lung disease*" OR "dust related lung disease*" AND<br>TOPIC: rehabilitat* OR telerehabilitat* OR "pulmonary rehabilitation" OR "home based rehabilitation" OR	No filters; search date: 29 June 2026	6

Database	Search Query (Keywords)	Filters Applied	Results
	"exercise training" OR "physical training" OR "respiratory training"<br>AND<br>TOPIC: digital OR remote OR telehealth OR telemedicine OR wearable* OR mobile OR sensor* OR application* OR smartphone* OR "web based" OR "technology assisted"		
SCOPUS	TITLE-ABS-KEY (pneumoconiosis OR silicosis OR asbestosis OR "coal worker pneumoconiosis" OR "coal workers pneumoconiosis" OR "mixed dust pneumoconiosis" OR "dust induced lung disease*" OR "dust related lung disease*")<br>AND<br>TITLE-ABS-KEY (telemonitoring OR "remote monitoring" OR wearable OR sensor* OR telemedicine OR telehealth OR "digital health" OR "home spirometry" OR oximetry OR "mobile app" OR "mobile apps" OR "mobile application*" OR IoT)	No filters; search date: 29 June 2026	148

Database	Search Query (Keywords)	Filters Applied	Results
	TITLE-ABS-KEY (pneumoconiosis OR silicosis OR asbestosis OR "coal workers pneumoconiosis" OR "coal worker pneumoconiosis" OR "mixed dust pneumoconiosis" OR "dust induced lung disease*" OR "dust related lung disease*") AND TITLE-ABS-KEY (rehabilitat* OR telerehabilitat* OR "pulmonary rehabilitation" OR "home based rehabilitation" OR "exercise training" OR "physical training" OR "respiratory training") AND TITLE-ABS-KEY (digital OR remote OR telehealth OR telemedicine OR wearable* OR mobile OR sensor* OR application* OR smartphone* OR "web based" OR "technology assisted")	No filters; search date: 29 June 2026	22
Total			383
